# Hybrid Xerogels: Study of the Sol-Gel Process and Local Structure by Vibrational Spectroscopy

**DOI:** 10.3390/polym13132082

**Published:** 2021-06-24

**Authors:** Guillermo Cruz-Quesada, Maialen Espinal-Viguri, María Victoria López-Ramón, Julián J. Garrido

**Affiliations:** 1 Departamento de Ciencias, Campus Arrosadía, Public University of Navarre (UPNA), 31006 Pamplona, Spain; guillermo.cruz@unavarra.es; 2 Institute for Advanced Materials and Mathematics (INAMAT^2^), Campus Arrosadía, Public University of Navarre (UPNA), 31006 Pamplona, Spain; 3 Departamento de Química Inorgánica y Orgánica, Facultad de Ciencias Experimentales, University of Jaén, 23071 Jaén, Spain; mvlro@ujaen.es

**Keywords:** ORMOSILs, xerogels, hybrid materials, chloroalkyltriethoxysilanes, inductive effect, (SiO)_x_ structures, FTIR

## Abstract

The properties of hybrid silica xerogels obtained by the sol-gel method are highly dependent on the precursor and the synthesis conditions. This study examines the influence of organic substituents of the precursor on the sol-gel process and determines the structure of the final materials in xerogels containing tetraethyl orthosilicate (TEOS) and alkyltriethoxysilane or chloroalkyltriethoxysilane at different molar percentages (RTEOS and ClRTEOS, R = methyl [M], ethyl [E], or propyl [P]). The intermolecular forces exerted by the organic moiety and the chlorine atom of the precursors were elucidated by comparing the sol-gel process between alkyl and chloroalkyl series. The microstructure of the resulting xerogels was explored in a structural theoretical study using Fourier transformed infrared spectroscopy and deconvolution methods, revealing the distribution of (SiO)_4_ and (SiO)_6_ rings in the silicon matrix of the hybrid xerogels. The results demonstrate that the alkyl chain and the chlorine atom of the precursor in these materials determines their inductive and steric effects on the sol-gel process and, therefore, their gelation times. Furthermore, the distribution of (SiO)_4_ and (SiO)_6_ rings was found to be consistent with the data from the X-ray diffraction spectra, which confirm that the local periodicity associated with four-fold rings increases with higher percentage of precursor. Both the sol-gel process and the ordered domains formed determine the final structure of these hybrid materials and, therefore, their properties and potential applications.

## 1. Introduction

Organically modified silicon xerogels (ORMOSILs) are attracting considerable interest for their application in new-generation technologies, being utilized in chemical and optical sensors [1,2,3,4,5,6], for catalysis [4,5], in coatings [6,7,8,9,10], for chromatography [11,12], and in pharmacy [13]. They have great chemical versatility and can be efficiently modified for specific applications due to their combination of highly varied mechanical, optical and textural properties [14].

The sol-gel method is the most widely used approach to the synthesis of hybrid silicon xerogels. It is based on co-condensation between monomers of traditional alkoxides (Si(OR)_4_) such as tetramethoxy- or tetraethoxysilane (TEOS and TMOS, respectively) and one or more mono-, di- or trialkylsilanes (R_x_Si(OR’)_4–x_) [15]. In hybrid xerogels, the addition of organic molecules or functional groups in the silica network restricts the three-dimensional growth of the material and blocks a condensation position, favoring the preferential formation of (SiO)_4_ and (SiO)_6_ rings in the amorphous structure of the silica materials [16,17] and even leading to the formation of structures with a higher degree of order [18]. However, although the sol-gel process has been widely studied [19], some questions have yet to be adequately settled. For instance, multiple reactions take place simultaneously in the process, making it difficult to extract information from experimental procedures [20]. To date, many studies have attempted to elucidate the influence of precursors on the hydrolysis, condensation reactions, and crosslinking by applying different characterization and analysis techniques, including gas chromatography (GC) [21], nuclear magnetic resonance ^29^Si NMR [22,23], Raman spectroscopy [24], and Fourier transform infrared spectroscopy (FTIR) [25,26]. Among these approaches, FTIR not only provides complementary information on the bonds and structures formed during the synthesis process but also, when combined with deconvolution methods, yields valuable data on the microstructure of siliceous materials [27,28,29]. Knowledge obtained by these means is of major interest because it allows for the prediction of: (i) the physical properties of xerogels derived from their structure [30]; (ii) the catalytic activity of metals supported in silica matrices [31]; and (iii) the correlation of the silica species in the membrane of a fiber optic sensor with its efficacy [32].

The ultimate application of these materials is as membranes in fiber optic sensors, on which the analyte is specifically and reversibly adsorbed. Physisorption of the analyte on the surface of the material generates a modification of the refractive index and produces a change in the reflected optical power, which determines the analyte concentration in the medium. For this reason, it is important to prepare materials with different porosities and surface chemistries in which the interaction between the membrane (chemical area of a sensor) and the analyte is specific and labile. To date, our research group has prepared membranes with hybrid xerogels obtained using different molar ratios of TEOS:RTEOS (R = methyl or phenyl) [33,34]. Given the results obtained and following this line of reasoning, the addition of a chlorine atom to the organic moiety of a silane emerged as a highly appealing approach because of the inductive effects of the chlorine, which generates a more active chemical surface and favors functionalization with other compounds. For these reasons, ClRTEOS precursors were used to synthesize three series of xerogels analogous to those prepared in previous studies [35].

The objective of this study was to determine the influence of the alkyl and chloroalkyl substituents of the precursors on: (i) the gelation time, and (ii) the microstructure of the synthesized materials, obtained by deconvolution of the FTIR spectra. The study used six series of hybrid xerogels prepared (in previous studies) with tetraethoxysilane (TEOS) and a chloroalkyl or alkyl precursor at different molar percentages (ClRTEOS or RTEOS: R = methyl [M], ethyl [E]; and propyl [P]) [35,36,37,38]. Theoretical study of the deconvolution of the band at 1300–980 cm^−1^ in the FTIR spectra yielded semi-quantitative information on the proportion of (SiO)_4_ and (SiO)_6_ rings related to periodic structural domains and amorphous silica, respectively. This allows the local internal order of materials to be determined and the influences of the organic substituent and chlorine atom to be predicted. The ultimate application of these materials is as membranes in fiber optic sensors, constituting the chemical area of the sensor, where the analyte is specifically and reversibly adsorbed. Physisorption of the analyte on the surface of the material generates a modification of the refractive index and produces a change in the reflected optical power, which determines the analyte concentration in the medium. This study is of crucial importance because: (i) precise knowledge of the gelation time is essential to effectively impregnate the fibers [33,34], and (ii) complete characterization of the xerogels allows prediction, to a large extent, of their properties and the a priori selection of the xerogel best suited to the characteristics of the analyte of interest [32].

## 2. Materials and Methods

### 2.1. Materials

The siliceous precursors TEOS (tetraethoxysilane, purity > 99%), ClMTEOS (chloromethyl)triethoxysilane, purity > 95%), and ClPTEOS ((3-chloropropyl)triethoxy silane, purity > 95%) were supplied by Sigma-Aldrich (San Luis, MO, USA), and ClETEOS ((2-Chloroethyl)triethoxysilane, purity > 95%) was obtained from Fluorochem (Glossop, UK). Absolute ethanol (Emsure^®^) and hydrochloric acid (HCl, 37% *w*/*w*) were purchased from Merck (Darmstad, Germany) and potassium bromide (FT-IR grade) from Sigma-Aldrich (San Luis, MO, USA). All chemicals were used without further purification.

### 2.2. Synthesis of the Xerogels

The three series of hybrid xerogels were prepared as previously described [35,36,37,38]. TEOS was mixed with ClRTEOS (R = Methyl [M], Ethyl [E] or propyl [P]) at different molar ratios, maintaining a constant (TEOS + RTEOS):ethanol:water ratio (1:4.75:5.5) throughout the series.

The reagent and solvent quantities were adjusted to obtain 20 mL of alcogels. First, TEOS and ClRTEOS were mixed in a 30 mL container (φ 3.5 cm, threaded plastic lid, Schrarlab, Barcelona, Spain). Absolute ethanol was then added, followed by the dropwise addition of Milli-Q grade water under magnetic stirring to facilitate miscibility. Once the pH of the mixtures remained unchanged (after ~10 min), an automatic burette (Tritino mod. 702 SM, Metrohm, Herisau, Switzerland) was used to set the pH at 4.5 (0.05 M solution of HCl), and the mixture was stirred for 10 min to ensure homogenization. The closed containers were placed in a thermostatized oven at 60 °C (J.P. Selecta S.A, Barcelona, Spain) until gelling, i.e., when the shape of the materials did not change when the container was tilted. Subsequently, 5 mL of ethanol were added to cure the alcogel at room temperature for one week. Next, the containers were opened and covered with paraflim^TM^, which was pierced with holes to facilitate evaporation of the solvent, and were then dried at room temperature under atmospheric pressure. The monolith was considered dried when no significant variation in its mass was observed. Finally, the xerogels were further dried (90 °C under vacuum) and then ground in an agate mortar.

### 2.3. Characterization

Infrared spectra were recorded using a FTIR spectrometer (Jasco, mod. 4700, Tokyo, Japan) at 25 scans and resolution of 4 cm^−1^. Compressed KBr tablets with two different concentrations of sample were prepared: (i) 0.6 mg sample in 200 mg KBr tablet for spectra in the range 2200–400 cm^−1^, thereby avoiding saturation of the Si-O-Si asymmetric stretching signal [39]; and (ii) 2 mg sample in 200 KBr tablet for spectra in the range 4000–2200 cm^−1^ for recording O-H and C-H bonds in greater detail. After their preparation, the tablets were dried over-night in an oven at 115 °C under vacuum to remove adsorbed water. Comparison between functional groups of the precursors in the pure reactant versus xerogel was carried out by using attenuated-total reflectance (ATR) to record the spectra directly from droplets of the precursors. The recorded transmittance of the samples was transformed into Kubelka-Munk units with the spectrometer software (Spectramanager, SMII FTIR Rev 216A ver2.15A) to allow their deconvolution (curve fitting) by a previously reported method [27]. Study parameters were four or five Gaussian-Lorentzian bands, with a maximum of 200 interactions, fixed baseline and >0.1 difference between experimental and synthetic spectra.

X-ray diffraction spectra were obtained at room temperature using a PANanalytical Empydrean XRD instrument (Empyrean, Almelo, The Netherlands) with graphite monochromator (at 45 KV and 40 mA) and copper rotating anode to select the CuK_α1/2_ wavelength at 1.54 nm. Measurements were performed in a stepped scan mode of 2 ≤ 2θ ≤ 50° in steps of 0.013° at a rate of 0.5 steps s^−1^ [40].

## 3. Results and Discussion

### 3.1. Influence of Organochlorine Substituents on Gelation Time

The gelation time is the period between the initial mixture of reagents and the formation of the gel; it comprises hydrolysis, condensation, and gelation (stabilization of colloids and crosslinking) stages, with gelation being the limiting stage [41]. Density functional theory (DFT) studies of TEOS in acidic media showed that the overall process of hydrolysis presents a pseudo-first order mechanism (SN_1_) with lesser energy barriers in comparison to condensation [20]. Moreover, each consecutive hydrolysis reaction presents a barrier with less energy, which is consistent with the hydrolysis rates observed in ^29^Si NMR experiments on various organoalkylsilanes [23]. The hydrolysis reactions of RTEOS are described in the following equations:

(1) $$\mathrm{RSi}{\left({\mathrm{OC}}_{2}H_{5}\right)}_{3}+H_{2}O\;\overset{k_{h1},\;\left[H_{3}O^{+}\right]}{\to }\;\mathrm{RSi}{\left({\mathrm{OC}}_{2}H_{5}\right)}_{2}\left(\mathrm{OH}\right)+{\;C}_{2}H_{5}\mathrm{OH},$$

(2) $$\mathrm{RSi}{\left({\mathrm{OC}}_{2}H_{5}\right)}_{2}\left(\mathrm{OH}\right)+{\;H}_{2}{O\;}_{\underset{k_{-h2},\;\left[H_{3}O^{+}\right]}{\leftarrow }}^{\overset{k_{h2},\;\left[H_{3}O^{+}\right]}{\to }}\mathrm{RSi}\left({\mathrm{OC}}_{2}H_{5}\right){\left(\mathrm{OH}\right)}_{2}+{\;C}_{2}H_{5}\mathrm{OH},$$

(3) $$\mathrm{RSi}\left({\mathrm{OC}}_{2}H_{5}\right){\left(\mathrm{OH}\right)}_{2}+{\;H}_{2}{O\;}_{\underset{k_{-h3},\;\left[H_{3}O^{+}\right]}{\leftarrow }}^{\overset{k_{h3},\;\left[H_{3}O^{+}\right]}{\to }}\mathrm{RSi}{\left(\mathrm{OH}\right)}_{3}+{\;C}_{2}H_{5}\mathrm{OH}.$$

Reaction rates in the acidic hydrolysis of triethoxysilanes depend on the competition between the steric hindrance and inductive effects of the organic moieties [16]. The reaction rate decreases with an increase in the volume of the organic moiety [42], while electron donating groups (^+^I) stabilize the pentacoordinated transition state and increase the electronic charge of the ethoxide in the hybrid precursor, facilitating the attack of the acidic proton (reactions 1–3) [20,43]. In previous studies, it was confirmed that the hydrolysis rates increase with a longer alkyl chain in the precursor due to the enhanced inductive effect of the moieties [23]. Given that chloroalkyltriethoxysilanes contain chlorine, an electron withdrawing group (^−^I), hydrolysis rates are expected to be lower than their non-chlorinated analogs, as can be deduced from the partial charges of the ethoxide groups (δ_OEt_ values of Table 1).

However, the condensation reactions determine the overall speed of the gelation process. The following equations exhibit the SN_2_ mechanism for the first and consecutive acidic condensation reactions of RTEOS:

(4) $$\mathrm{RSi}{\left(\mathrm{OH}\right)}_{3}+\;\mathrm{RSi}{\left(\mathrm{OH}\right)}_{3}\overset{k_{c1},\;\left[H_{3}O^{+}\right]}{\to }\mathrm{OH}-\mathrm{RSi}\left(\mathrm{OH}\right)-O-\mathrm{RSi}\left(\mathrm{OH}\right)-\mathrm{OH}+{\;H}_{2}O,$$

(5) $$\mathrm{RSi}{\left(\mathrm{OH}\right)}_{3}+{\left(\mathrm{OH}\right)}_{m}{\left(\mathrm{RSiORSi}\right)}_{n}\;\overset{k_{c2},\;\left[H_{3}O^{+}\right]}{\to }{\left(\mathrm{OH}\right)}_{m-1}{\left(\mathrm{RSiORSi}\right)}_{n+1}+{\;H}_{2}O.$$

In contrast to the hydrolysis reactions (Reactions 1–3), the first condensation between hydrolyzed molecules is kinetically favored, leading initially to the formation of long and slightly branched chains and subsequently to intra-molecular condensations and cyclization [16,45,46]. The successive condensations form the polymeric network and the first colloidal particles (φ = 10–100 nm), which give rise to a spontaneous and homogeneous nucleation process when their critical radius is reached. It should be noted that gelation of the hybrid material is slower because triethoxysilanes have only three potential directions of polymerization. Unlike in the hydrolysis process, where the more the substituent of the precursor is withdrawn, the more favored is the condensation, because it favors nucleophilic attack on the silicon atom of a neighboring molecule. In this way, the condensation rate increases in the order MTEOS > ETEOS > PTEOS for the alkyl series and ClMTEOS > ClETEOS > ClPTEOS for the chloroalkyl series. In addition, chloroalkyl groups restrict the crosslinking between oligomers due to electrostatic repulsions, which destabilize the colloids and prevent the gelling process above a given molar percentage. Thus, it was possible to synthesize hybrid xerogels with molar percentages of up to 35%, 25% and 25% for the chloroalkyl series (ClMTEOS, ClETEOS and ClPTEOS respectively, Figure S1), while the molar percentages were substantially higher for the alkyl series [36,37,38].

**Figure 1:**
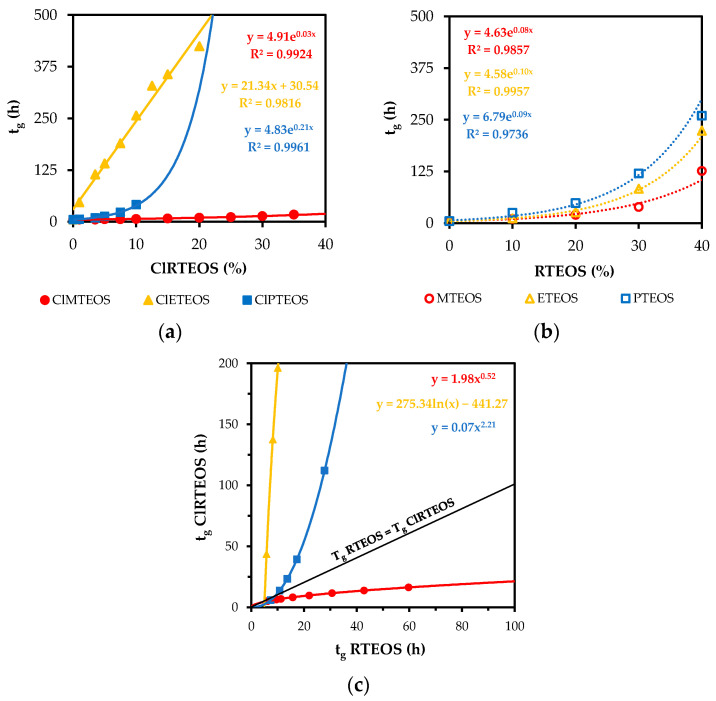
(**a**) t_g_ as a function of the percentage of precursor in the TEOS:ClRTEOS series, (**b**) t_g_ as a function of the percentage of t precursor in the TEOS:RTEOS series, and (**c**) t_g_ of the ClRTEOS versus RTEOS series.

Hence, with regard to the gelation times of the synthesized materials, there is a balance between the inductive/steric effects of the organic substituents of the precursor and the electrostatic effect between colloids. This electrostatic effect was a determining factor in the gelation times of chloroalkyl xerogels with a high percentage of precursor, due to the chlorine atom. The corresponding data are displayed in Figure 1a, which depicts the variation in gelation times of the chloroalkyl xerogels as a function of the molar percentage of precursor. It should be noted that gelation times for percentages higher than 10% have been omitted for the ClPTEOS series because they do not follow the trend observed for 0 to 10 molar percentages due to the limitations imposed by steric and electrostatic factors. In all cases, an increase in the molar percentage lengthens the gelation time, and this trend is also observed in xerogels prepared with analogous alkyl precursors, as shown in Figure 1b [36,37,38]. Figure 1c compares gelation times between the chloroalkyl xerogels and their non-chlorinated analogs, showing that the effect of the chlorine atom is more pronounced in the ClETEOS and ClPTEOS series, besides being opposite to the effect in the ClMTEOS series. This is because the alkyl chain is longer in the ClETEOS and ClPTEOS, increasing the steric effect and the repulsions between colloids created by the chlorine atom, which markedly lengthens the gelation time in comparison to their analogous non-chlorinated series. The chain is shorter in the ClMTEOS series; therefore, the steric effect is less pronounced and the repulsion between colloids is lower, favoring crosslinking in comparison to the longer chain chloroalkyl series. Furthermore, the withdrawing effect of the chlorine atom on the adjacent silicon atom is maximized, favoring condensation. Accordingly, the gelation times for the CIMTEOS series are reduced with respect to the analogous non-chlorinated series.

It should be noted that t_g_ values are higher for the ClETEOS series than for the other two chloroalkyl series and fit a linear rather than exponential trend (Figure 1a). This is because the length of the chloroethyl chain is not as short as in the ClMTEOS series or as long as in the ClPTEOS series, which has greater flexibility and freedom of movement. As demonstrated below, the behavior of this substituent is different because its size and nature necessarily place it in the network at fixed positions, minimizing repulsions. The lack of freedom of movement hinders and slows crosslinking, markedly increasing gelation times and forming periodic structures that create ordered domains in the silica network, as explained below.

### 3.2. Study of the Local Structure of Hybrid Xerogels Using FTIR and XRD

**Figure 2:**
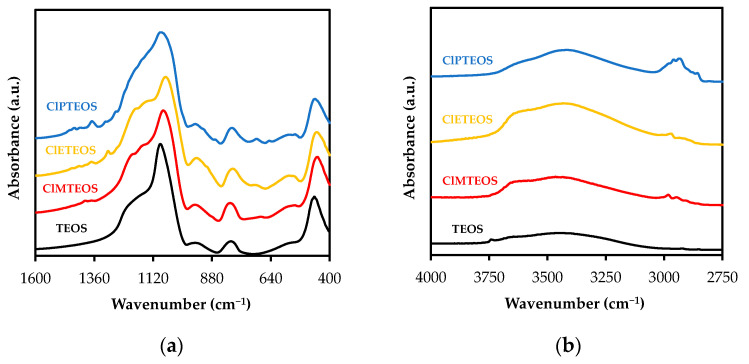
FTIR spectra of TEOS xerogel and TEOS:ClRTEOS hybrids (molar ratio, 20%): (**a**) range from 1600 to 400 cm^−1^, and (**b**) range from 4000 to 2750 cm^−1^.

Figure 2 depicts, as an example, the FTIR spectra of the three chloroalkyl series with 20% molar percentage of the precursor in two wavelength ranges: (i) 4000–2750 cm^−1^ and (ii) 1600–400 cm^−1^. The spectra obtained for the xerogels prepared with different molar percentages of chloroalkyl precursor and the different modes of vibration are reported in Supplementary Material with corresponding citations from the literature (Figure S2, Table S1).

Figure 2a depicts the characteristic FTIR bands of the silica lattice: (i) rocking of O-Si-O (ρ O-Si-O) at 455 cm^−1^, (ii) symmetric stretching vibration of Si-O-Si (ν_s_ Si-O-Si) at 800 cm^−1^, (iii) stretching of the Si-O bond belonging to surface silanes (ν_s_ Si-OH) at 955 cm^−1^, (iv) asymmetric stretching vibration Si-O-Si at 1090 cm^−1^ (ν_as_ Si-O-Si), and (v) a broad and intense shoulder at 1200 cm^−1^ related to various modes of vibration of the Si-O-Si bonds [47]. Additionally, materials synthesized using silsesquioxanes typically show a set of Si-O-Si vibrations belonging to different structures: (i) bicyclic species at 1007 cm^−1^, (ii) linear species at 1020 cm^−1^, and (iii) cyclic species at 1050 cm^−1^ [48]. As can be observed in the figure, bands associated with bicyclic and linear species do not appear to overlap with the band at 1090 cm^−1^, indicating that these structures are present in lower proportions than are the cyclic structures. Figure 2b displays the bands of isolated (or non-interacting) surface silanols and silanols interacting by hydrogen bonds at 3660 cm^−1^ and 3450 cm^−1^, respectively. These bands, which are also characteristic of silicon xerogels, indicate the hydrophobic or hydrophilic nature of the material [49]. In addition to the aforementioned bands, a shoulder is observed at 550 cm^−1^ in all spectra, associated with the presence of 4-membered siloxane rings, (SiO)_4_ [50,51], which is consistent with the aforementioned assumption of the predominance of cyclic species.

The presence of organic groups in these hybrid materials is revealed by the bond vibrations of the alkyl chain: (i) C-H, (ii) C-C; and (iii) C-Cl. In Figure 2b, the characteristic bands of symmetric and asymmetric stretching of C-H are clearly observed between 2890 and 2975 cm^−1^, showing an increase in intensity with longer chain length from being practically null in TEOS to being readily identifiable in ClPTEOS. The same can be seen in Figure 2a for the region between 1250 and 1400 cm^−1^, which shows the bands of deformation modes related to C-H bonds (δ C-H) [52]. The symmetric stretching band C-C (ν_s_ C-C) of chloroethyl was detected in ClETEOS series at 900 cm^−1^ [53], while two well-differentiated bands were detected at 920 and 870 cm^−1^ in the ClPTEOS series, corresponding to the C-C bond contiguous to the chlorine and silicon atom, respectively (Table S1). This figure also depicts C-Cl stretching vibration bands (ν C-Cl) in the region between 750 and 650 cm^−1^ [53,54], with an increased intensity at higher molar percentages of ClRTEOS, as expected (Figure S2). It should be noted that it was not possible to detect the spectral band of the wagging vibration of the Si-CH_2_ bond (ω C-H) in the region between 1300 and 980 cm^−1^ and the band corresponding to the out-of-plane deformation of the Si-C bond due to overlap with the frequencies of the bending vibration of the Si-O bond at 800 cm^−1^. However, both vibrations can be clearly observed in the attenuated total reflection (ATR) spectrum of the pure precursor (Figure S3).

**Figure 3:**
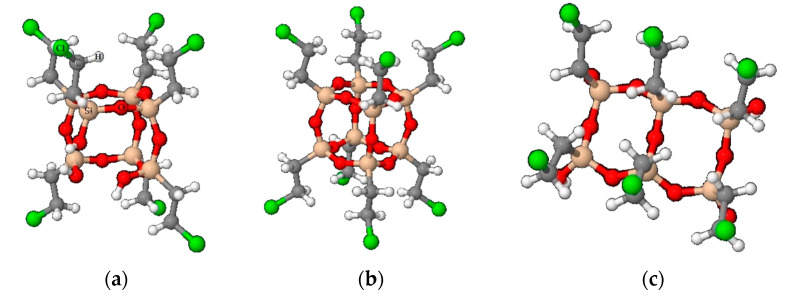
Ordered structures in the silica matrix built by (SiO)_4_ rings in ClETEOS as an example: (**a**) open cage (T_7_), (**b**) cage (T_8_), and (**c**) short ladders.

In the alkyl series previously studied by our research group (RTEOS:TEOS, R = M, E or P), a splitting of the band is observed at high percentages of alkyl precursor, moving the original band to lower frequency values due to the inductive effect of alkyl groups [36,37,38]. In the ETEOS series, for example, the band at 1092 cm^−1^ shifts to 1043 cm^−1^ and a new band appears at 1128 cm^−1^, increasing its relative absorbance with higher molar percentage of ETEOS. The appearance of this band is related to the presence of highly symmetric structures within the amorphous silica matrix (Figure 3). These structures comprise four-membered silicon rings (SiO)_4_ and are described as close or open cages (T_8_ and T_7_, respectively) and short ladders [54,55]. The intensity of this new band increases with higher molar percentage of the chloroalkyl precursor due to stabilization of the rings (SiO)_4_ and minimization of the electrostatic repulsions produced by the chlorine atoms. In the chloroalkyl series, the asymmetric Si-O-Si vibration also shifts towards lower frequency values with increased proportion of the precursor. The bands are not resolved in this case because the molar percentage reached is lower than in the alkyl series; instead, shoulders are observed at around 1200 cm^−1^.

**Figure 4:**
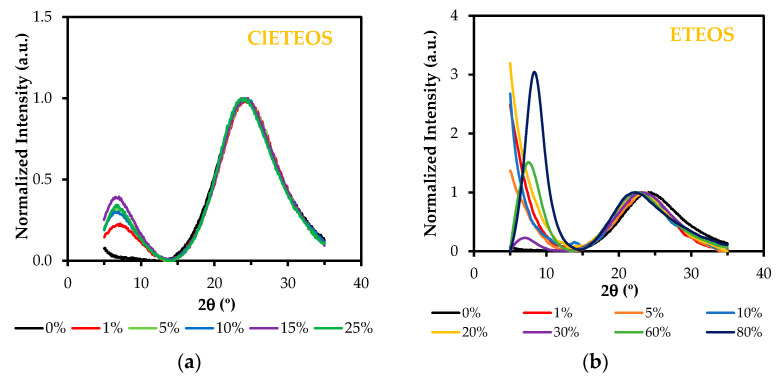
X-ray diffraction spectra of the hybrid xerogels at different molar percentages (normalized with respect to the band 2θ ~4°: (**a**) ClETEOS:TEOS, and (**b**) ETEOS:TEOS.

The presence of these structures in both RTEOS:TEOS and ClRTEOS:TEOS hybrid xerogels is consistent with the X-ray diffraction spectra obtained for these materials. Figure 4 depicts the X-ray diffraction spectra of the ClETEOS:TEOS series (the only series that follows a linear trend in gelation times). In addition to the characteristic diffraction peak of amorphous silica (2θ ~ 24) [56,57], another peak can be observed at 2θ < 10° with only 1% of precursor. This peak is related to cage-like and ladder-like oligomeric species that form ordered domains within the amorphous matrix of the xerogel [58,59], which, according to computational chemistry studies, are the most thermodynamically stable structures for MTEOS:TEOS hybrid xerogels at pH 4.5 [60]. In the analogous ETEOS series, this diffraction peak is detected above 30% ETEOS and is consistent with the mass spectrometry results, which indicate the presence of ladder-like structures within the silica matrix [37]. Consequently, the appearance of this peak at much lower molar percentages in the ClETEOS series can be explained by the steric and electronic properties of the chloroethyl substituent, conferring rigidity or freedom of movement restriction to the organic moiety. This compromises the crosslinking and encourages the early formation of ordered periodic domains in the 3D structure. The difficult cross-linking of the colloids or oligomers would explain the anomalous trends in gelation times for this series. The spectra of the series of hybrid xerogels and the corresponding data are exhibited in Supplementary Material (Figure S4, Table S2).

### 3.3. Spectral Deconvolution of the 1300–700 cm^−1^ Region

**Figure 5:**
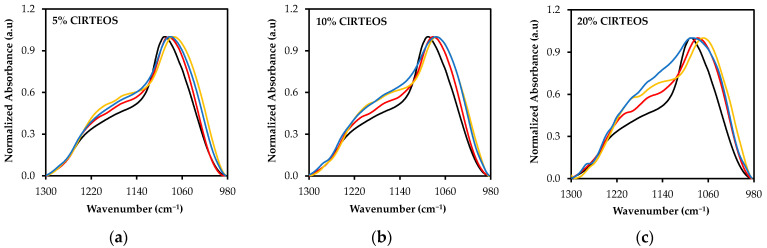
Normalized FTIR spectra of the 1300–980 cm^−1^ region of the TEOS xerogel (black), and those of the three chloroalkyl series at different molar ratios: (**a**) 5%, (**b**) 10%, and (**c**) 20%. (ClMTEOS (red), ClETEOS (yellow), and ClPTEOS (blue)).

Figure 5 depicts the ν_as_ Si-O-Si band at 1090 cm^−1^ and the associated shoulder at around 1200 cm^−1^. It shows the shift at lower frequencies of the band at 1090 cm^−1^ with higher molar percentages of the precursor as well as the overlapping bands derived from the shoulder at 1200 cm^−1^. These emerging bands are attributable to the different structures that make up the siloxane bonds, mostly forming rings of four or six silicon atoms, (SiO)_4_ and (SiO)_6_ respectively. The relative abundance of these rings depends on the nature and molar ratio of the precursor: four-fold rings are the predominant species in xerogels and are thermodynamically favored in the oligomerization of TEOS at acid pH through cyclodimerization processes [45,47], whereas six-fold rings are kinetically favored over four-membered rings and constitute the main structures of silicates and amorphous silica [17,61,62]. In the deconvolution study, each structure is associated with two optical modes of ν_as_ Si-O-Si vibration in the FTIR spectra due to the Coulomb interactions: (i) transverse mode (TO), between 1100 and 1000 cm^−1^; and (ii) longitudinal mode (LO), between 1250 and 1100 cm^−1^ [63].

**Figure 6:**
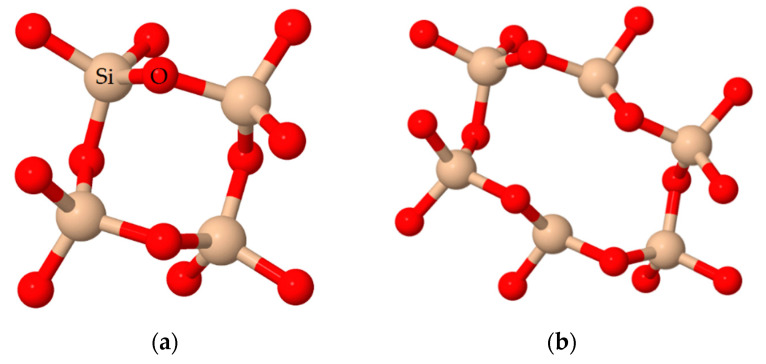
Predominant structures within the silica matrix of a xerogel prepared with TEOS: (**a**) (SiO)_4_ rings, and (**b**) (SiO)_6_ rings.

The changes in these structures as the precursor increases and their relative proportions were studied by deconvolution of the FTIR spectra in the range 1300–980 cm^−1^, using the non-linear least-squares method to obtain the Gaussian-Lorentzian components. The different distances and degrees of torsion of Si-O-Si bonds in the structures, along with the optical modes, predict four components of ν_as_ Si-O-Si in the studied range: (i) TO_4_ and LO_4_ for (SiO)_4_ rings, and (ii) TO_6_ and LO_6_ for (SiO)_6_ rings. Therefore, four components were introduced in the software for deconvolution of the reference material spectrum (100% TEOS Xerogel, Figure 2). The resulting optimized synthetic spectrum was composed of bells with maxima at 1214, 1143, 1092 and 1078 cm^−1^, assigned to LO6, LO_4_, TO_4_ and TO_6_, respectively. This assignation takes account of: (i) the aforementioned frequency ranges of the optical modes; (ii) the predominance of (SiO)_4_ species evidenced by the shoulder at 550 cm^−1^ in the spectra, and (iii) the broader limiting frequencies in both optical modes for six-fold rings, related to their less tensioned nature (Figure 6) [27,47,64].

**Figure 7:**
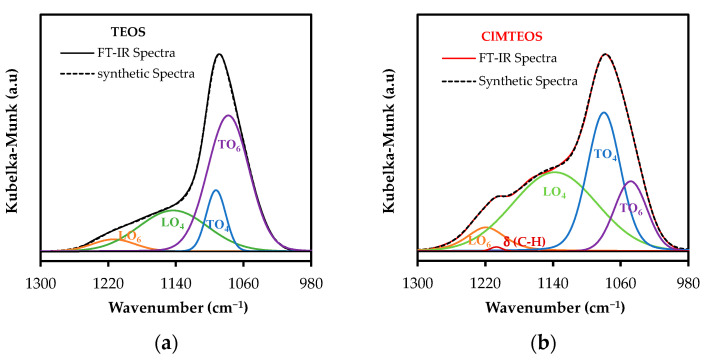

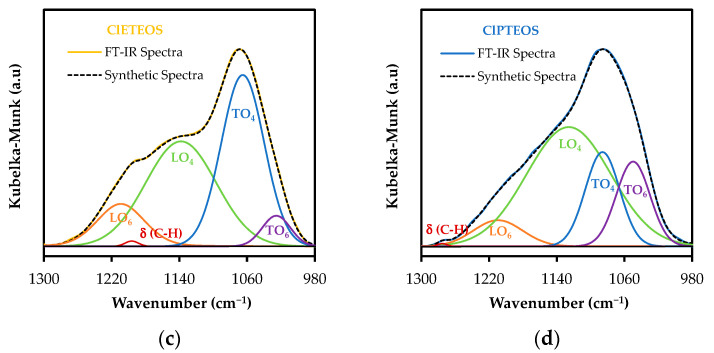
Deconvolution and least-squares adjustment of the FTIR spectra of the xerogels: (**a**) Reference material (100% TEOS), (**b**) 20% ClMTEOS, (**c**) 20% ClETEOS, and (**d**) 20% ClPTEOS.

Spectra of the RTEOS:TEOS and ClRTEOS:TEOS series were deconvoluted using the aforementioned frequencies obtained from the reference material (LO_6_, LO_4_, TO_4_ and TO_6_) and the frequencies of C-H wagging vibrations corresponding to the organic moiety of each precursor (Table S1, supporting material). By way of example, Figure 7 depicts the synthetic spectra derived from the bell-shaped curves obtained for TEOS (Figure 7a) and the organochlorinated xerogels with a 20% molar percentage of precursor (actual FTIR spectra shown in Figure 5c).

Based on the integrated areas of the Gaussian-Lorentzian bells, Equations (6) and (7) were calculated to determine the relative abundance of (SiO)_6_ and (SiO)_4_ rings (Tables S3 and S4 for the ClRTEOS:TEOS and RTEOS:TEOS series, respectively).

(6) $${\left(\mathrm{SiO}\right)}_{6},\;\%=\frac{A\left({\mathrm{LO}}_{6}\right)+A\left({\mathrm{TO}}_{6}\right)}{A\left({\mathrm{LO}}_{4}\right)+A\left({\mathrm{TO}}_{4}\right)+A\left({\mathrm{LO}}_{6}\right)+A\left({\mathrm{TO}}_{6}\right)}\;100,$$

(7) $${\left(\mathrm{SiO}\right)}_{4},\;\%=\frac{A\left({\mathrm{LO}}_{4}\right)+A\left({\mathrm{TO}}_{4}\right)}{A\left({\mathrm{LO}}_{4}\right)+A\left({\mathrm{TO}}_{4}\right)+A\left({\mathrm{LO}}_{6}\right)+A\left({\mathrm{TO}}_{6}\right)}\;100,$$

where A(LO_6_) is the area of the band at 1220 cm^−1^, A(LO_4_) is the area of the band at 1150 cm^−1^; A(TO_4_) is the area of the band at 1070 cm^−1^ and A(TO_6_) the area of the band at 1050 cm^−1^.

**Figure 8:**
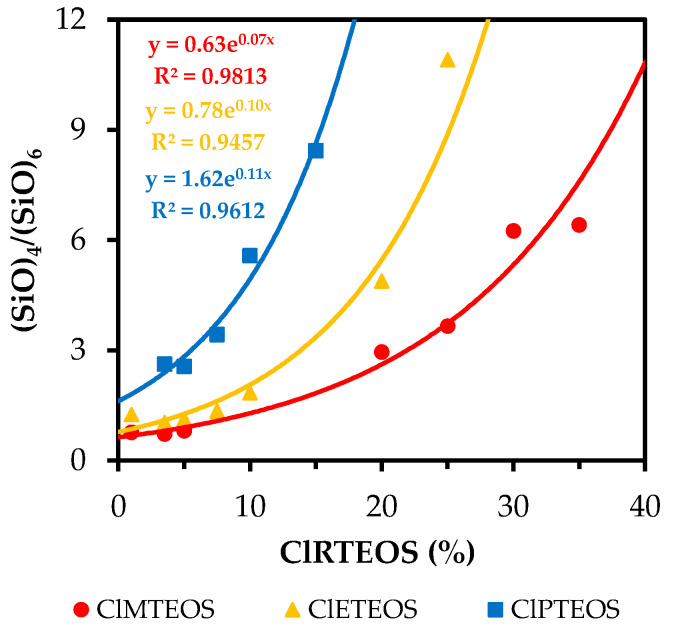
Variation in (SiO)_4_/(SiO)_6_ as a function of the molar percentage of precursor in ClRTEOS:TEOS xerogels.

Figure 8 graphically depicts the (SiO)_4_/(SiO)_6_ ratio as a function of the molar percentage of chloroalkyl precursor (data obtained from Tables S3 and S4, see Supplementary Material). It shows that the formation of (SiO)_4_ rings is more favored with longer chloroalkyl chain for a given molar percentage of precursor. The (SiO)_4_/(SiO)_6_ ratio increases exponentially with a higher percentage of precursor in the three series. These trends are not expected, because the entry of organic molecules or substituents into the network should produce an increase in the proportion of (SiO)_6_ species, which minimizes steric tension, increases the volume, and markedly reduces the density (Table S5). Accordingly, Fidalgo, A. et al. found that the proportion of (SiO)_6_ rings increases with higher molar percentage of RTEOS (R = M, E and P), reaching an abundance of almost 85% for samples with a precursor percentage of 75% [27]. However, the synthesis of these materials was performed in basic medium, explaining the greater abundance of six-membered rings from crosslinking between more branched chains. In the case of the present xerogels, the higher proportion of (SiO)_4_ rings in the chloroalkyl series would be related not only to their synthesis in acidic medium but also to the formation of ordered domains at very low molar percentages of precursor. The data in Table S3 (Supplementary Material) for the three chloroalkyl series show that the contribution of the LO_4_ band (attributable to the total percentage of (SiO)_4_ rings) increases with higher percentage of precursor. This is a relevant finding, given that this band is also associated with the presence of ordered structures in the literature [5,51,65]. Its presence is closely related to a decrease in the degree of crosslinking in the xerogel, in agreement with the shifts at lower wavelengths of the TO_4_ band, which is associated with the silicon network [13]. Another factor that supports the formation of these ordered structures is the decrease in the intensity of the LO_6_ band (see Table S3), because this bands is generally associated not only with (SiO)_6_ rings but also with the non-silica porous skeleton [66].

The way in which the chlorine atom affects the ring distribution was studied in greater depth by performing interpolations using the equations of the exponential curves shown in Figure 8 and Figure S5 ((SiO)_4_/(SiO)_6_ vs. %RTEOS). These equations were used to obtain (SiO)_4_/(SiO)_6_ ratios for percentages of the precursor in the range 1–35% in both the chlorinated series and their non-chlorinated analogs (the data used are exhibited in Table S6 of Supplementary Material).

**Figure 9:**
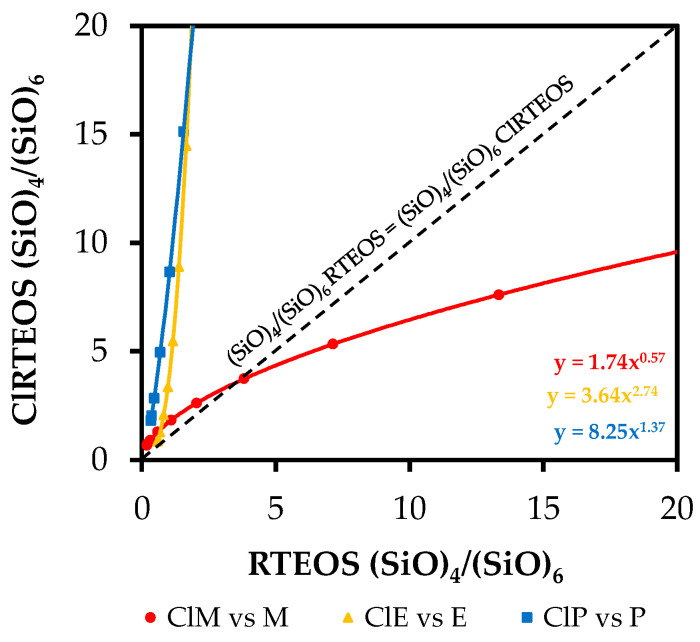
Variation in the proportion of structures (SiO)_4_/(SiO)_6_ of the chloroalkyl series with respect to the alkyl series at different molar percentages of precursor.

Figure 9 compares (SiO)_4_/(SiO)_6_ ratios between the ClRTEOS:TEOS series and the RTEOS:TEOS series to show how the chlorine atom affects the relative proportion of species.

A discontinuous black straight line has been added in this figure to depict the hypothetical case in which the ratio of (SiO)_4_/(SiO)_6_ rings is the same for the alkyl and chloroalkyl series at the same molar percentage of precursor, which would indicate that the chlorine atom in the ClRTEOS precursor has no effect on the (SiO)_4_:(SiO)_6_ ratio with respect to its counterpart, RTEOS. Above this line, the effect of the chlorine on the (SiO)_4_:(SiO)_6_ ratio is positive, favoring the formation of (SiO)_4_ rings. Below this line, the effect of the chlorine is negative, favoring the formation of (SiO)_6_ structures. The figure shows that this ratio is lower in the ClMTEOS than in the MTEOS series. This trend is consistent with the behavior observed in Figure 1c, which shows that the ClMTEOS series has shorter gelation times, closely related to the size of the chloroalkyl chain. This not only fails to produce a significant steric or electrostatic effect to disfavor the formation of (SiO)_6_ rings (kinetically favored species), but also accelerates condensation due to the inductive effect of the chlorine, which reduces gelation times and gives rise to lower (SiO)_4_/(SiO)_6_ ratios. Unlike the ClMTEOS series, the (SiO)_4_/(SiO)_6_ values for the ClETEOS and ClPTEOS series are higher than those of their analogs throughout the region, which is in turn consistent with the longer gelation times displayed in Figure 1c. In these two series, the steric factor and the electrostatic repulsions between colloids significantly increase the crosslinking time, disfavoring (SiO)_6_ ring formation. The slope of these curves is much steeper than the slope observed for ClMTEOS series, implying that the addition of a higher percentage of precursor increases the influence exerted by the chlorine atom on the structure of the synthesized materials, which is maximized in ClPTEOS:TEOS, the series with the longest chain. It should be noted that, although the (SiO)_4_/(SiO)_6_ ratio is slightly lower in the ClETEOS versus ClPTEOS series, the former has longer gelation times and peaks at small angles (10° < 2θ) in all X-ray diffraction spectra. According to these findings, the chloroethyl moiety in this material is more efficient in directing the formation of ordered domains and nanostructuring the material, even when it has fewer (SiO)_4_ rings (related to periodic box or ladder-type structures), consistent with the results of all techniques used to characterize these hybrid materials [35].

## 4. Conclusions

In general, an increase in the molar percentage of the precursor (ClRTEOS and RTEOS) translates into an increase in gelation times due to conjugation of the steric and inductive effects of the organic substituents. The gelation times of the chloroalkyl series are longer than those of their analogous alkyl series for long alkyl chains (ClETEOS and ClPTEOS), although they are slightly shorter for the shortest alkyl chain (ClMTEOS). This is explained by the maximization of the inductive effect of the chlorine atom due to its proximity to the silicon atom, which exerts a positive influence on the condensation and crosslinking reactions, the slowest step in the sol-gel process. On the other hand, a larger number of carbons in the alkyl chain reduces the inductive effect of the chlorine on the silicon atom and increases the steric effect exerted by the chain to form the silica network; this effect is maximized in the series bearing ethyl, chloroethyl, propyl and chloropropyl groups. Unexpectedly, the ClETEOS series have the highest gelation times, explained by: (i) the lack of flexibility of the chloroethyl group due to the steric tension caused by its size; (ii) the electrostatic repulsions exerted by chlorine, forcing the formation of kinetically disfavored structures in order to minimize its energy; and (iii) the X-ray diffraction spectra of this series (1–25%), which reveal maximum diffraction at a small angle (2θ < 10°), consistent with the presence in their structure of periodic domains that require more time for their formation. The FTIR spectra of each hybrid xerogel yielded data for a semi-quantitative determination of the proportions of (SiO)_4_ and (SiO)_6_ rings by deconvolution methods. These findings reveal a competitive process between the two species that is dependent on the precursor and its molar ratio, observing similar trends to those obtained for the gelation times. In the ClMTEOS series, the presence of the chlorine atom favors the formation of six-fold rings. An opposite trend is observed in the ClETEOS and ClPTEOS series, favoring the formation of 4-fold rings; in addition, the chlorine atom exerts a stronger influence in the ClETEOS and ClPTEOS series, consistent with their longer gelation times and the presence of periodic structures. According to these results, the substitution of a hydrogen atom by a halogen functional group (e.g., chlorine) in hybrid xerogels produces relevant changes in its microstructure due to the intermolecular forces generated by the chlorine atom in the sol-gel process.

## Figures and Tables

**Table 1:** Electronegativities and partial charges of the chloroalkyl precursors and their alkyl analogs calculated from the Pauli electronegativities and application of the equations of Livage and Henry [44].

Precursor	R	X	δ_Si_	δ_R_	δ_OEt_
TEOS	OC_2_H_5_	2.32	0.32	-	−0.08
MTEOS	CH_3_	2.29	0.31	0.20	−0.17
ETEOS	C_2_H_5_	2.29	0.31	0.28	−0.20
PTEOS	C_3_H_7_	2.28	0.30	0.35	−0.22
ClMTEOS	CH_2_Cl	2.32	0.32	−0.09	−0.08
ClETEOS	C_2_H_4_Cl	2.31	0.32	0.02	−0.11
ClPTEOS	C_3_H_6_Cl	2.30	0.31	0.11	−0.14

X, molecule electronegativity; δ, partial charge.

## Data Availability

The data presented in this study are available on request from the corresponding author.

## Supplementary Materials

The following are available online at https://www.mdpi.com/article/10.3390/polym13132082/s1, Figure S1: Images of the synthesized xerogels in which a change in morphology is observed with an increasing percentage of organic precursors: (**a**) TEOS, (**b**–**d**) ClMTEOS, (**e**–**g**) ClETEOS, and (**h**–**j**) ClPTEOS; Figure S2: FTIR spectra of the ClRTEOS:TEOS xerogels in the ranges 4000–2750 and 1600–400 cm^−1^: (**a**,**b**) ClMTEOS, (**c**,**d**) ClETEOS, and (**e**,**f**) ClPTEOS; Figure S3: FTIR spectra of the precursors (TEOS, ClMTEOS, ClETEOS and ClPTEOS) in the ranges: (**a**) 4000–2750 cm^−1^ and (**b**) 1600–400 cm^−1^; Figure S4: X-ray diffraction spectra of ClRTEOS:TEOS and RTEOS:TEOS xerogels: (**a**) ClMTEOS, (**b**) MTEOS, (**c**) ClPTEOS, and (**d**) PTEOS; Figure S5: (SiO)_4_/(SiO)_6_ ratio with respect to the molar percentage of RTEOS:TEOS xerogels. Table S1: FTIR spectra band assignment; Table S2: Bragg angles (2θ), band area (A), and bond distance (d1 and d2 (nm)) calculated from the X-ray diffraction peaks for the xerogels synthesized with ClMTEOS, MTEOS, ClPTEOS, and PTEOS; Table S3: Relative areas obtained from deconvolution of the FTIR spectra and percentages of (SiO)_4_ and (SiO)_6_ in the ClRTEOS:TEOS series; Table S4: Relative areas obtained from deconvolution of the FTIR spectra and percentages of (SiO)_4_ and (SiO)_6_ in the RTEOS:TEOS series; Table S5: Helium density of the hybrid xerogels; Table S6: experimental data of (SiO)_4_/(SiO)_6_ and equations used for Figure 8 and Table 1.
