# Supplementary material for: Hybrid Xerogels: Study of the Sol-Gel Process and Local Structure by Vibrational Spectroscopy
Source: Polymers (Basel). 2021 Jun 24;13(13):2082. doi: 10.3390/polym13132082 (PMC8271438; doi:10.3390/polym13132082)
Supplement: Supplementary file 1 [file polymers-13-02082-s001.zip › polymers-1262807-supplementary.pdf]

SUPPORTING MATERIAL

Hybrid Xerogels: Study on the Sol-Gel Process and the Local Structure by Vibrational Spectroscopy

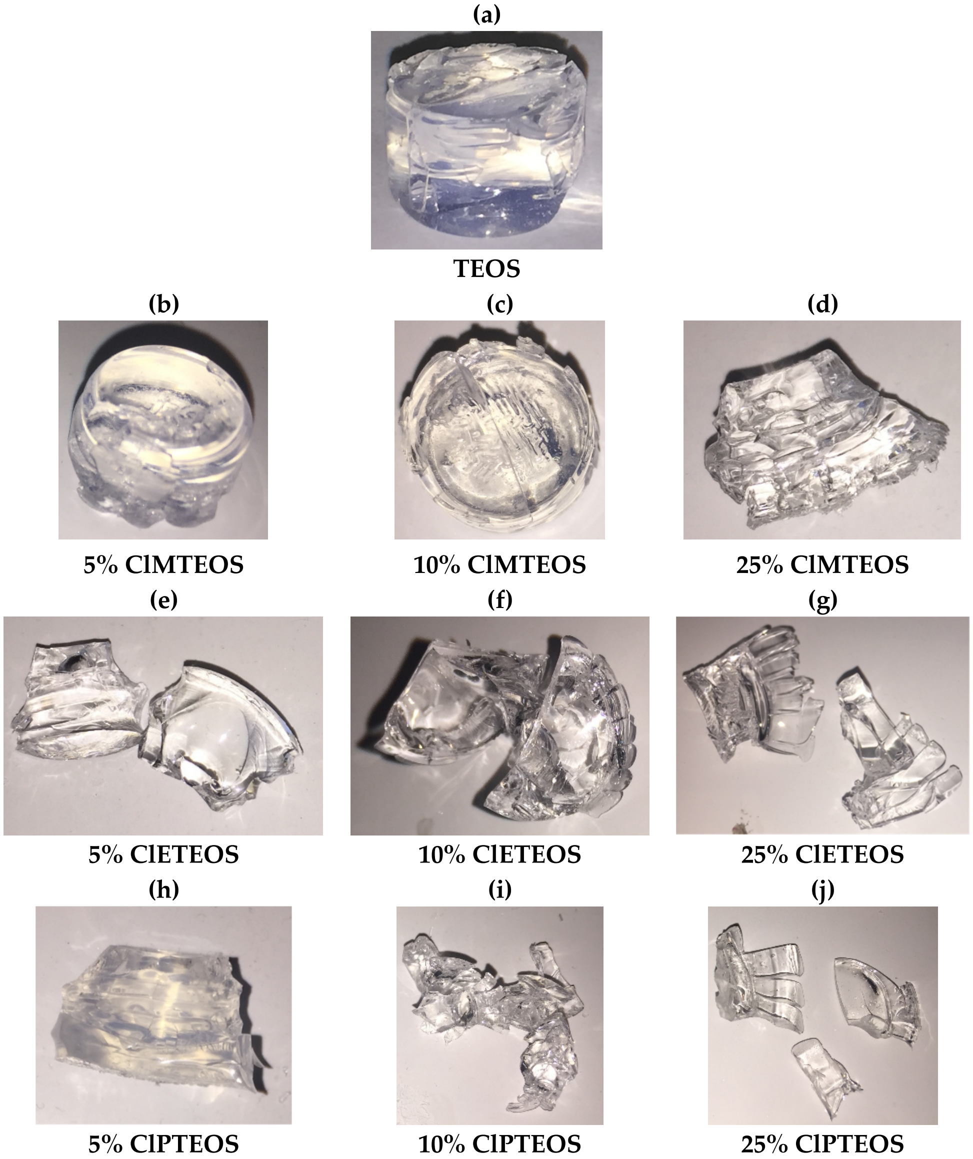

Figure S1: Images of the synthesized xerogels where a change in morphology is observed with an increasing percentage of organic precursors: a) TEOS, b-d) ClMTEOS, e-g) ClETEOS, and h-j) ClPTEOS.

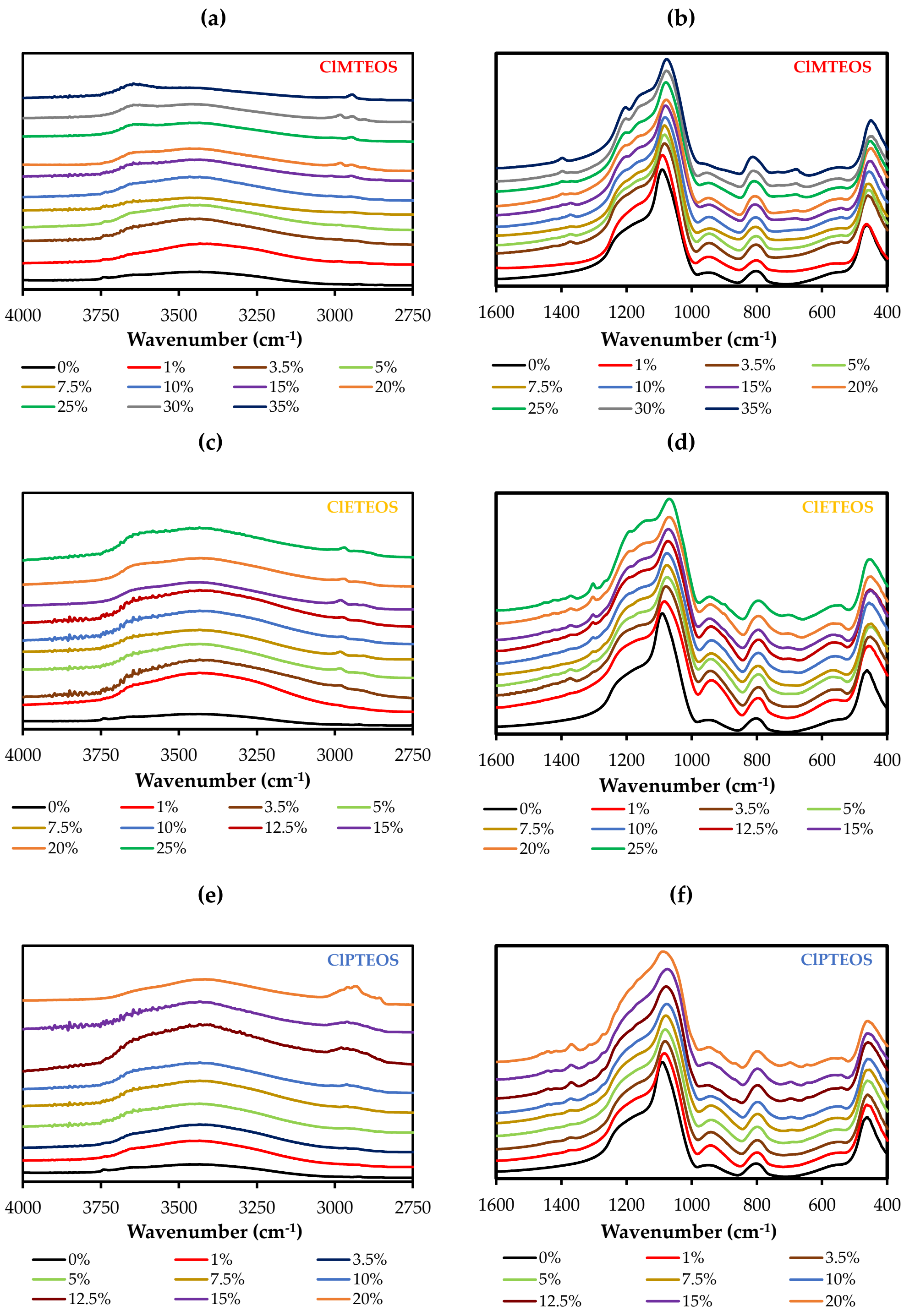

Figure S2: FTIR spectra of the ClRTEOS:TEOS xerogels in the ranges of 4000-2750 and 1600-400 cm^{-1}: a,b) ClMTEOS, c,d) ClETEOS, and e,f) ClPTEOS.

Table S1: FTIR spectra band assignement.

Wave number (cm^{-1})	Type of Vibration	Structural Unit	References
3645	ν (OH-H)	SiO-H	[50]
3465	ν (OH-H)	SiO-H (H-Bridge)	[50]
2980	ν_{as} (C-H)	CH_3/CH_2-Cl	[35-38]
2945	ν_{as} (C-H)	CH_3/CH_2-Cl	[35-38]
2928	ν_{as} (C-H)	CH_2	[35-38]
2900	ν_s (C-H)	CH_3/ CH_2-Cl	[35-38]
2850	ν_s (C-H)	CH_2	[35-38]
1640	δ (O-H)	H_2O	[52]
1480	τ (C-H)	CH_3/CH_2-Cl	[52]
1445	τ (C-H)	R-CH_2-R	[52]
1420	τ (C-H)	Si-CH_2-R	[52]
1395	δ (C-H)	CH_3/CH_2-Cl	[52-54]
1370	δ (C-H	CH_3/CH_2-R	[52,53]
1310	δ (C-H)	Si-CH_2-R	[52-54]
1270	ω (C-H)	CH_3/-CH_2-Cl	[52-54]
1180	ω (C-H)	Si-CH_2-Cl	[53,54]
1200	ν_{as} (Si-O-Si mode LO)	≡Si-O-Si≡	[27,64]
1150	ν_{as} (Si-O-Si)	≡Si-O-Si≡ (T_8)	[51]
1090	ν_{as} (Si-O-Si mode TO)	≡Si-O-Si≡	[27,50,64]
955	ν (Si-O)	≡Si-O-H/ ≡Si-O^-	[27,53]
915	ν (C-C)	Si-CH_2-CH_2-R	[53]
865	ν (C-C)	R-CH2-CH2-Cl	[53]
800	ν_s (Si-O)	≡Si-O-Si≡	[50]
735	ν_{as} C-Cl	CH_2-Cl	[51,64]

680	ν_s C-Cl	CH_2-Cl	[52,54]
560	ν Si-O	Si-O_2 (T_4)	[51]
455	ρ Si-O	O-Si-O	[50,53]

ν, stretching vibration; ν_s, symmetric stretching; ν_{as}, antisymmetric stretching; δ, bending; τ, twisting; ω, wagging; ρ, rocking; LO, longitudinal optical; TO, transverse optical; T_8, cubic octamer; T_4, four-fold siloxane rings.

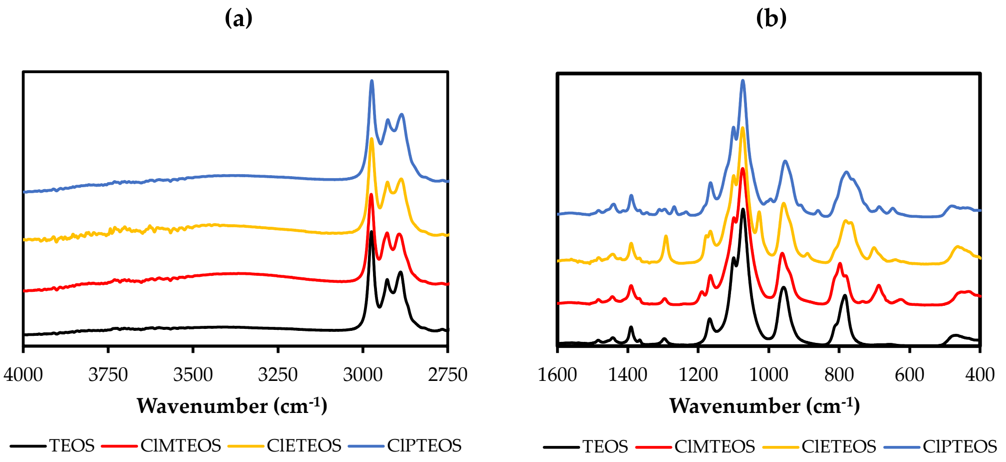

Figure S3: FTIR-ATR spectra of the precursors (TEOS, ClMTEOS, ClETEOS and ClPTEOS) in the ranges: a) 4000-2750 cm^{-1} and b) 1600-400 cm^{-1}.

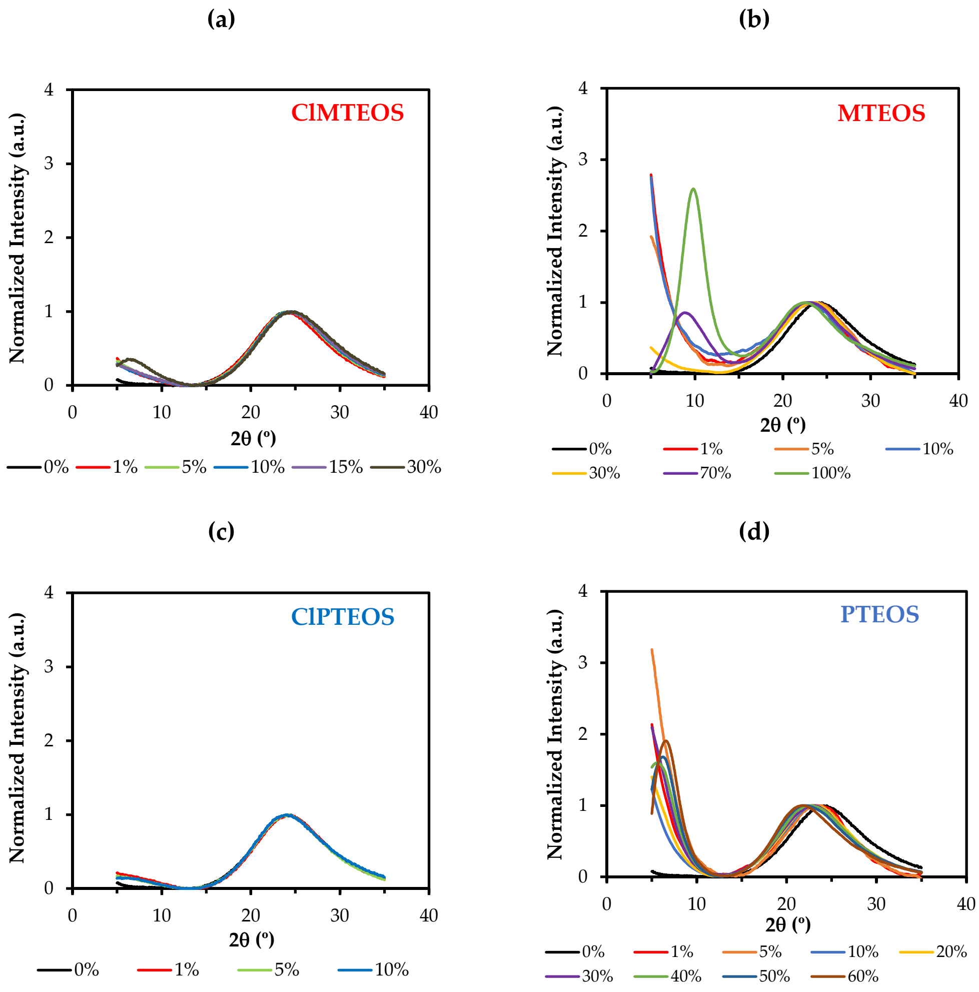

Figure S4: X-Ray diffraction spectra of ClRTEOS:TEOS and RTEOS:TEOS xerogels: a) ClMTEOS, b) MTEOS, c) ClPTEOS, and d) PTEOS [35-38].

Table S2: Bragg angles (2θ), band area (A), and bond distance (d1 and d2 (nm)) calculated from the X-Ray diffraction peaks for the xerogels synthesized with ClMTEOS, MTEOS, ClPTEOS and PTEOS [35-38].

RTEOS	RTEOS (%)	Peak 2θ < 10°		Peak 10° > 2θ < 30°	
		2θ (°)	d (nm)	2θ (°)	d (nm)
TEOS	0	*	*	24.16	0.368
RTEOS					
MTEOS	1	*	*	22.99	0.387
	5	*	*	23.85	0.373
	10	*	*	22.72	0.391
	30	*	*	23.51	0.378
	70	8.81	1	22.82	0.39
	100	9.83	0.9	22.46	0.396
ETEOS	1	*	*	22.52	0.395
	5	*	*	23.58	0.377
	10	*	*	22.86	0.389
	20	*	*	23.02	0.386
	30	7.07	1.25	23.18	0.384
	60	7.49	1.18	22.5	0.395
	80	8.36	1.06	22.3	0.399
PTEOS	1	*	*	23.38	0.38
	5	*	*	23.96	0.371
	10	*	*	22.88	0.389
	20	*	*	23	0.387
	30	*	*	23.12	0.385
	40	5.6	1.58	22.67	0.392
	50	6.23	1.42	21.8	0.408
	60	6.59	1.34	21.86	0.407
ClRTEOS					
ClMTEOS	1	*	*	24.26	0.367
	5	*	*	24.3	0.366
	10	*	*	24.54	0.363
	15	*	*	24.42	0.365
	30	6.52	1.35	24.78	0.359
ClETEOS	1	7.17	1.23	24.56	0.362
	5	6.92	1.28	24.3	0.366
	10	6.76	1.31	24.14	0.369
	15	6.76	1.31	24.22	0.367
	25	6.76	1.31	23.9	0.372
ClPTEOS	1	*	*	24.22	0.367
	5	*	*	23.98	0.371
	10	5.8	1.52	24.06	0.37

* non detected

Table S3: Relative areas obtained from the deconvolution of the FTIR spectra and $(SiO)_4$ and $(SiO)_6$ percentages for ClRTEOS:TEOS series [35].

ClRTEOS	ClRTEOS molar ratio (%)	(LO)$_6$ ν (cm^{-1})	(LO)$_6$ (%)	(LO)$_4$ ν (cm^{-1})	(LO)$_4$ (%)	(TO)$_4$ ν (cm^{-1})	(TO)$_4$ (%)	(TO)$_6$ ν (cm^{-1})	(TO)$_6$ (%)	(SiO)$_4$* (%)	(SiO)$_6$* (%)
TEOS	0	1214	4.9	1143	27.3	1093	11.6	1078	56.1	39.0	61.0
ClMTEOS	1	1216	5.0	1145	32.7	1092	10.8	1082	51.5	43.5	56.5
	3.5	1219	6.5	1145	36.3	1089	5.7	1077	51.5	42.0	58.0
	5	1221	6.4	1147	36.5	1091	8.2	1076	48.9	44.7	55.3
	7.5	1202	14.4	1126	32.1	1087	32.5	1061	21.0	64.6	35.4
	10	1218	8.3	1138	42.5	1077	42.9	1043	6.4	85.3	14.7
	15	1215	9.2	1137	38.2	1082	37.7	1051	14.9	75.9	24.1
	20	1219	9.4	1138	42.6	1080	32.0	1049	15.8	74.6	25.2
	25	1218	9.8	1140	39.4	1079	39.1	1048	11.7	78.5	21.5
	30	1226	4.7	1136	52.5	1074	33.7	1040	9.1	86.2	13.8
	35	1223	4.5	1136	48.6	1076	37.9	1044	9.0	86.5	13.5
ClETEOS	1	1216	11.3	1139	37.2	1090	18.3	1058	33.1	55.6	44.4
	3.5	1214	11.2	1137	36.4	1089	14.5	1059	37.9	50.9	49.1
	5	1212	10.7	1136	39.0	1086	13.8	1058	36.5	52.8	47.2
	7.5	1210	11.5	1135	40.3	1083	17.1	1053	31.1	57.4	42.6
	10	1210	12.4	1136	38.5	1078	26.5	1044	22.6	64.9	35.1
	15	1208	12.8	1134	40.8	1076	28.1	1043	18.3	68.9	31.1
	20	1209	12.1	1138	41.7	1065	41.3	1025	4.9	83.0	17.0
	25	1211	7.0	1138	52.2	1062	39.4	1021	1.4	91.6	8.4
ClPTEOS	1	1220	6.9	1146	34.8	1089	6.0	1074	52.2	40.9	59.1
	3.5	1214	8.9	1140	33.3	1085	39.1	1055	18.7	72.4	27.6
	5	1216	9.0	1135	40.8	1083	31.2	1049	19.0	71.9	28.1
	7.5	1214	9.1	1133	44.0	1079	33.5	1044	13.4	77.4	22.6
	10	1219	6.6	1142	42.1	1075	42.7	1039	8.6	84.8	15.2
	15	1216	7.7	1145	38.3	1071	51.1	1040	2.9	89.4	10.6
	20	1209	7.4	1126	57.6	1086	18.6	1050	16.4	76.2	23.8
	25	1199	11.0	1124	45.7	1096	9.7	1064	33.5	55.4	44.6

* Proportion of Rings calculated with equations (1) and (2).

Table S4: Relative areas obtained from the deconvolution of the FTIR spectra and $(SiO)_4$ and $(SiO)_6$ percentages for RTEOS:TEOS series [36-38].

RTEOS	RTEOS molar ratio (%)	(LO)$_6$ ν (cm^{-1})	(LO)$_6$ (%)	(LO)$_4$ ν (cm^{-1})	(LO)$_4$ (%)	(TO)$_4$ ν (cm^{-1})	(TO)$_4$ (%)	(TO)$_6$ ν (cm^{-1})	(TO)$_6$ (%)	(SiO)$_4$* (%)	(SiO)$_6$* (%)
TEOS	0	1214	4.9	1143	27.3	1093	11.6	1078	56.1	39.0	61.0
MTEOS	10	1228	6.9	1147	32.8	1094	5.3	1071	55.0	38.1	61.9
	20	1224	4.1	1142	38.5	1088	29.5	1053	27.8	68.1	31.9
	30	1221	3.7	1137	44.1	1066	41.1	1026	11.1	85.1	14.9
	40	1216	2.8	1139	44.9	1059	51.7	1020	0.6	96.6	3.4
	50	1212	4.0	1137	43.6	1067	30.6	1034	21.8	74.2	25.8
ETEOS	10	1228	4.6	1149	34.5	1094	7.2	1071	53.6	41.8	58.2
	20	1209	6.6	1117	38.9	1080	45.7	1051	8.8	84.6	15.4
	30	1216	2.1	1131	54.4	1062	40.9	1044	2.6	95.3	4.7
	40	1212	3.2	1119	68.0	1089	9.1	1052	19.7	77.1	22.9
	50	1209	3.7	1125	62.3	1066	25.1	1041	8.9	87.4	12.6
PTEOS	10	1224	4.5	1134	39.7	1084	43.1	1052	12.8	82.7	17.3
	20	1203	12.4	1132	28.7	1080	34.9	1048	24.0	63.6	36.4
	30	1212	6.2	1128	44.5	1080	19.7	1050	29.6	64.2	35.8
	40	1204	9.1	1134	34.7	1069	43.0	1046	13.2	77.8	22.2
	50	1199	3.2	1122	60.4	1054	35.7	1027	0.6	96.2	3.8

* Proportion of Rings calculated with equations (1) and (2).

Table S5: Helium density for the hybrid xerogels [35].

RTEOS	RTEOS Molar ratio (%)	Helium density g/cm^3
**TEOS**	0	1.96
**ClMTEOS**	5	1.94
	10	1.91
	20	1.9
**ClETEOS**	5	1.88
	10	1.85
	20	1.79
**ClPTEOS**	5	1.85
	10	1.8
**MTEOS**	5	1.82
	10	1.8
	20	1.72
**ETEOS**	5	1.8
	10	1.76
	20	1.61
**PTEOS**	5	1.86
	10	1.76
	20	1.58

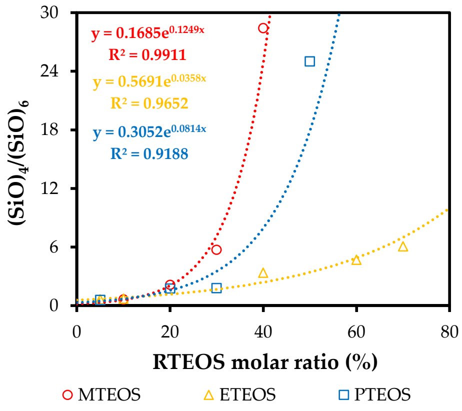

Figure S5: $(SiO)_4/(SiO)_6$ ratio with respect to the molar percentage of RTEOS:TEOS xerogel [36-38].

Table S6: experimental data of $(SiO)_4/(SiO)_6$ and equations used for Figure 9 and Figure S5

Precursor	Precursor molar ratio (%)	$(SiO)_4$ (%)	$(SiO)_6$ (%)	$(SiO)_4/(SiO)_6$	Fitting Ecuation	Fitting R^2
**RTEOS**						
MTEOS	10	38.1	61.9	0.6	$y = 0.1685e^{0.1249x}$	0.9911
	20	68.1	31.9	2.1		
	30	85.1	14.9	5.7		
	40	96.6	3.4	28.4		
ETEOS	5	39.6	60.4	0.7	$y = 0.5691e^{0.0358x}$	0.9652
	10	41.8	58.2	0.7		
	40	77.1	22.9	3.4		
	60	82.4	17.6	4.7		
	70	85.9	14.1	6.1		
PTEOS	5	36.4	63.6	0.6	$y = 0.3052e^{0.0814x}$	0.9188
	20	63.6	36.4	1.7		
	30	64.2	35.8	1.8		
	50	96.2	3.8	25.0		
**ClRTEOS**						
ClMTEOS	1	43.5	56.5	0.8	$y = 0.6317e^{0.0711x}$	0.9813
	3.5	42.0	58.0	0.7		
	5	44.7	55.3	0.8		
	20	74.6	25.2	3.0		
	25	78.5	21.5	3.7		
	30	86.2	13.8	6.3		
	35	86.5	13.5	6.4		
ClETEOS	1	55.6	44.4	1.3	$y = 0.7789e^{0.0974x}$	0.9457
	3.5	50.9	49.1	1.0		
	5	52.8	47.2	1.1		
	7.5	57.4	42.6	1.3		
	10	64.9	35.1	1.8		
	20	83.0	17.0	4.9		
	25	91.6	8.4	10.9		
ClPTEOS	3.5	72.4	27.6	2.6	$y = 1.6182e^{0.1117x}$	0.9612
	5	71.9	28.1	2.6		
	7.5	77.4	22.6	3.4		
	10	84.8	15.2	5.6		
	15	89.4	10.6	8.4		

References

35. Cruz-quesada, G.; Espinal-viguri, M.; Garrido, J. Novel Organochlorinated Xerogels : From Microporous Materials to Ordered Domains. . *Polymers* **2021**, *13(9)*, 1415, doi: 10.3390/polym13091415.

36. Rios, X.; Moriones, P.; Echeverría, J.C.; Luquín, A.; Laguna, M.; Garrido, J.J. Characterisation of hybrid xerogels synthesised in acid media using methyltriethoxysilane (MTEOS) and tetraethoxysilane (TEOS) as precursors. *Adsorption* **2011**, *17*, 583–593, doi:10.1007/s10450-011-9331-9.

37. Rios, X.; Moriones, P.; Echeverría, J.C.; Luquin, A.; Laguna, M.; Garrido, J.J. Ethyl group as matrix modifier and inducer of ordered domains in hybrid xerogels synthesised in acidic media using ethyltriethoxysilane (ETEOS) and tetraethoxysilane (TEOS) as precursors. *Mater. Chem. Phys.* **2013**, *141*, 166–174, doi:10.1016/j.matchemphys.2013.04.042.

38. Moriones, P. Sintesis y caracterización de xerogeles silíceos híbridos (RTEOS/TEOS; R= P, Ph). Universidad Publica de Navarra: Pamplona, Spain, 2015. Available online: https://academica-e.unavarra.es/handle/2454/20351(accessed on 14 November 2020).

27. Fidalgo, A.; Ciriminna, R.; Ilharco, L.M.; Pagliaro, M. Role of the alkyl-alkoxide precursor on the structure and catalytic properties of hybrid sol-gel catalysts. *Chem. Mater.* **2005**, *17*, 6686–6694, doi:10.1021/cm051954x.
50. Innocenzi, P. Infrared spectroscopy of sol–gel derived silica-based films: a spectra-microstructure overview. J. Non. Cryst. Solids 2003, 316, 309–319, doi:https://doi.org/10.1016/S0022-3093(02)01637-X.
51. Handke, M.; Kowalewska, A. Siloxane and silsesquioxane molecules - Precursors for silicate materials. Spectrochim. Acta - Part A Mol. Biomol. Spectrosc. 2011, 79, 749–757, doi:10.1016/j.saa.2010.08.049.
52. Coates, J. Encyclopedia of Analytical Chemistry -IInterpretation of Infrared Spectra, A Practical Approach. *Encycl. Anal. Chem.* **2004**, 1–23. doi: 10.1002/9780470027318.a0815.
53. Launer, P.J.; Arkles, B. Infrared Analysis of Orgaonsilicon Compounds. *Silicon Compd. Silanes Silicones (3rd Ed.* **2013**, 175–178.
54. Chen, G.; Zhou, Y.; Wang, X.; Li, J.; Xue, S.; Liu, Y.; Wang, Q.; Wang, J. Construction of porous cationic frameworks by crosslinking polyhedral oligomeric silsesquioxane units with N-heterocyclic linkers. *Sci. Rep.* **2015**, *5*, 1–14, doi:10.1038/srep11236.
64. Caresani, J.R.; Lattuada, R.M.; Radtke, C.; Dos Santos, J.H.Z. Attempts made to heterogenize MAO via encapsulation within silica through a non-hydrolytic sol-gel process. *Powder Technol.* **2014**, *252*, 56–64, doi:10.1016/j.powtec.2013.10.015.
